# Hepatocyte Growth Factor: A Marker of Cardiac Function, Mortality, and Disease Subtype in Cardiac Amyloidosis

**DOI:** 10.1016/j.jacadv.2025.101828

**Published:** 2025-06-25

**Authors:** Margrethe Flesvig Holt, Annika E. Michelsen, August Flø, Yusuf Khan, Vilde Karoline Bjørnø, Mona Skjelland, Vigdis Bjerkeli, Benedicte Paus, John-Peder Escobar Kvitting, Bente Halvorsen, Tale Norbye Wien, Melinda Raki, Lars Gullestad, Pål Aukrust, Kaspar Broch, Thor Ueland, Einar Gude

**Affiliations:** a Department of Cardiology, Oslo University Hospital, Rikshospitalet, Oslo, Norway; b Research Institute of Internal Medicine, Oslo University Hospital, Rikshospitalet, Oslo, Norway; c Institute of Clinical Medicine, Faculty of Medicine, University of Oslo, Oslo, Norway; d Clinic for Head, Neck and Reconstructive Surgery, Oslo University Hospital, Rikshospitalet, Oslo, Norway; e Department of Neurology, Oslo University Hospital, Rikshospitalet, Oslo, Norway; f Department of Medical Genetics, Oslo University Hospital, Oslo, Norway; g Department of Cardiothoracic Surgery, Oslo University Hospital, Rikshospitalet, Oslo, Norway; h Department of Internal Medicine and Department of Medical Research, Bærum Hospital, Vestre Viken Hospital Trust, Drammen, Norway; i Department of Pathology, Oslo University Hospital, Rikshospitalet, Oslo, Norway; j Section of Clinical Immunology and Infectious Diseases, Oslo University Hospital Rikshospitalet, Oslo, Norway; k Thrombosis Research Center (TREC), Division of Internal Medicine, University Hospital of North Norway, Tromsø, Norway

**Keywords:** amyloid light chain, ATTR-CM, biomarker, cardiac amyloidosis, hepatocyte growth factor

## Abstract

**Background:**

It is important to reduce diagnostic delays for patients with cardiac amyloidosis (CA). Plasma biomarkers could streamline the diagnostic process and enhance prognostic accuracy.

**Objectives:**

The authors aimed to identify circulating biomarkers capable of differentiating patients with CA from patients with heart failure (HF) and no amyloidosis. Additionally, we assessed whether these markers were associated with patient outcomes.

**Methods:**

We performed focused protein screening in 12 patients with transthyretin CA, 5 patients with HF, and 16 healthy controls (HCs). To validate the findings, we used immunoassays to measure levels of differentially regulated proteins in a larger sample of 86 patients with transthyretin CA, 15 patients with light-chain CA, 16 patients with HF, and HCs. We compared protein levels between groups using multivariable general linear models. Associations between protein levels and all-cause mortality were assessed by receiver operating characteristic analysis.

**Results:**

We identified 99 candidate proteins by proteomic screening. In the validation sample, 4 of these markers were higher in CA than in HCs. Levels of C-X-C motif chemokine ligand 9 and hepatocyte growth factor (HGF) were also higher in CA than in HF. HGF correlated with measures of cardiac function in patients with transthyretin and light chain CA. HGF had a good discriminatory ability for predicting all-cause mortality (area under the curve = 0.80, *P* < 0.001), similar to those of N-terminal pro-B-type natriuretic peptide and troponin T.

**Conclusions:**

Plasma HGF is a promising screening tool for CA. Higher levels of HGF are associated with more severe HF and worse prognosis in patients with CA.

Cardiac amyloidosis (CA) is an infiltrative cardiomyopathy caused by deposition and aggregation of amyloid fibrils in the extracellular space.^1^ The progressive accumulation of amyloid leads to increased wall thickness, ventricular stiffness, arrhythmias, and heart failure (HF).^2^^,^^3^ Most cases of CA are caused by the deposition of misfolded transthyretin or immunoglobulin light chains.^3^ Transthyretin is a plasma protein produced primarily by the liver. It circulates as a tetramer. However, when the tetramer dissociates, transthyretin monomers may degenerate and coalesce to form oligomers and protofibrils, which can elongate into insoluble amyloid fibrils.^4^ In contrast, light chain amyloidosis (AL) results from the deposition of excess free light chains produced by an abnormal plasma cell clone. While AL is a distinctive condition, it is closely related to multiple myeloma.^4^^,^^5^

CA is a rare cause of HF that shares several clinical, echocardiographic, and biochemical features with more common causes of HF.^3^^,^^6^ However, distinguishing between CA and other, more prevalent heart diseases remains challenging. The diagnosis of CA requires additional diagnostic tests.^3^^,^^7^ Diagnostic delay is a common issue. Patients frequently see multiple physicians before receiving a correct diagnosis.^8^, ^9^, ^10^ The perceived rarity of the condition, along with nonspecific clinical findings and the need for specialized diagnostic tests, can contribute to these delays.

The prognosis is worse for patients with CA compared to patients with other causes of HF.^4^^,^^11^^,^^12^ However, targeted therapy is available, and novel disease-modifying drugs are under development.^3^^,^^13^, ^14^, ^15^ These innovations are expected to improve the prognosis for patients with CA. Disease-modifying treatment provides the most benefit when administered early, highlighting the importance of timely diagnosis.^15^, ^16^, ^17^ As treatment options for CA continue to expand, there is an increasing need for tools to aid prognostication and risk stratification.

Plasma biomarkers can help facilitate the diagnostic process and offer prognostic information. However, researchers have not identified a specific marker for CA. Furthermore, the pathogenic mechanisms in CA remain only partially understood. Several mechanisms may contribute to organ dysfunction, including intracellular signaling cascades triggered by protein aggregation and remodeling of the extracellular matrix. Further research into the molecular pathways in CA could reveal novel targets for therapy as well as diagnostic and prognostic biomarkers.

The aim of this study was to identify circulating biomarkers that identify patients with CA, particularly transthyretin amyloid cardiomyopathy (ATTR-CM), and to explore the extent to which these biomarkers are associated with disease progression and outcomes.

## Methods

### Study sample

We prospectively enrolled patients who were referred to our tertiary care center for the evaluation of suspected CA or for the initiation of disease-modifying treatment for established ATTR-CM. Study participants underwent clinical examination, electrocardiograms, echocardiograms, right heart catheterization with biopsy, bone scintigraphy, and laboratory tests. Transthoracic echocardiography was performed using commercially available ultrasound scanners as part of the routine clinical evaluation. We performed strain analyses irrespective of heart rhythm. Right heart catheterization was performed as described by Ravnestad et al.^18^ We measured cardiac output by thermodilution and recorded right atrial pressure, right ventricular pressures, mean pulmonary artery pressure, and pulmonary arterial wedge pressure. Pulmonary vascular resistance (in Wood units) was calculated as the difference between the mean pulmonary artery pressure and pulmonary arterial wedge pressure divided by cardiac output. Pulmonary hypertension was defined by a mean pulmonary artery pressure >20 mm Hg.^19^

CA was diagnosed based on the criteria outlined in the position paper by the European Society of Cardiology’s Working Group on Myocardial and Pericardial Diseases.^3^ Cardiac retention on bone scintigraphy was scored on planar images using the Perugini grading scale, which compares myocardial bone tracer uptake with rib tracer uptake on a scale from 0 (no cardiac uptake) to 3 (cardiac uptake higher than rib uptake).^20^ Transthyretin CA was diagnosed by biopsy or by cardiac uptake grade 2 or 3 in the absence of AL as determined by laboratory testing.^3^ Cardiac amyloid AL was diagnosed by cardiac biopsy.^3^

For comparison, we enrolled patients with HF who had a cardiac biopsy negative for amyloid. We also collected samples from self-reported healthy controls (HCs). The Regional Committee for Medical Research Ethics South East Norway approved the study (REK No. 2018/1846). Written informed consent was obtained from all participants.

### Plasma sample preparation

Venous blood was drawn into tubes containing ethylenediaminetetraacetic acid, placed on melting ice, and centrifuged at 2,000*g* at 4 °C for 20 minutes to gain platelet-poor plasma. We stored plasma in multiple aliquots at −80 °C.

### Targeted protein biomarker screening

We did focused protein screening in plasma using the commercially available Olink Target 96 panels Cardiovascular II, Cardiometabolic, and Inflammation from Olink Proteomics at the Proteomics Core Facility at the Department of Immunology, University of Oslo/Oslo University Hospital. These panels contain a mixture of known and novel cardiovascular and inflammatory proteins that are potentially linked to HF. Each panel contains 92 oligonucleotide-labeled antibody probe pairs that bind to their respective targets in plasma. This allows hybridization of the DNA-tag sequences and proximity extension with subsequent real-time polymerase chain reaction amplification. A set of internal and external quality controls were used to standardize and normalize the cycle threshold data. The raw data were log2 transformed and returned as normalized protein expression values (www.olink.com).

### Validation of proteomic data

To validate the results of the protein biomarker screening, we used multiplexing bead technology or enzyme immunoassays (EIAs) to measure levels of circulating proteins in a larger sample of patients. We analyzed dipeptidyl peptidase 4 (DPP4), hepatocyte growth factor (HGF), C-X-C motif chemokine ligand 9/monokine induced by gamma interferon (CXCL9/MIG), galectin-9, and serpin A12 in plasma by multiplex suspension technology with multiplexable beads from R&D Systems. The samples were analyzed on a Bio-Plex200 (BioRad) and quantified with the BioPlex Manager Software (BioRad). We used antibodies from R&D Systems to measure plasma levels of transforming growth factor beta receptor 3 (TGFβR3) and decorin (DCN) by EIA. Apolipoprotein M was measured by EIA using antibodies (M5 as capture and biotinylated M7 as detection) from MabTech and parallel-diluted reference plasma as standard (Accuclot TM Reference Plasma, Normal, A-7432). EIAs were run in a 384-well format using a CyBio SELMA pipetting robot (Analytik Jena) in combination with a BioTek (Agilent Technologies) dispenser/washer. Absorption was read at 450 nm with a wavelength correction set to 540 nm using a plate reader (BioTek). Intra- and inter-assay variation was <10% for all assays.

### Statistical analysis

Baseline characteristics are expressed as mean ± SD, median (IQR) or numbers (percentage), as appropriate. To compare between-group differences in demographics, we used chi-square, 1-way analysis of variance, or Kruskal-Wallis tests with post hoc tests. Proteomic data analysis was performed using R (version 4.4.0) and R Studio (version 2023.03.0). We performed one-way analysis of variance using the OlinkAnalyze R package (version 3.8.2) to compare protein expression in the screening sample. We adjusted all *P* values for multiple comparisons using the Benjamini-Hochberg procedure to control the false discovery rate (FDR) at 5%. Various R packages were employed for data visualization. We used Tukey plots to visualize marker levels.

To compare protein levels between groups in the validation cohort, we used a multivariable general linear model (GLM) with proteins as target, diagnostic group as fixed factor, and age, sex, and body mass index as covariables. Protein levels were skewed and log10 transformed prior to GLM analysis. However, we displayed the untransformed protein levels using box-and-whisker plots. Post hoc comparisons were adjusted using the Sidak method. Correlations were assessed by Spearman rho.

The associations between protein levels and all-cause mortality were assessed by receiver operating characteristic (ROC) analysis. Associations were further visualized by Kaplan-Meier analysis, with a cutoff identified by Youden index. We assessed the effect of the protein biomarkers on mortality in a Cox regression model. The model was adjusted for estimated glomerular filtration rate, N-terminal pro-B-type natriuretic peptide (NT-proBNP), cardiac troponin T (cTnT), left ventricular ejection fraction, and NYHA functional class. These established risk markers were added to the model one by one because of the low number of outcomes for this analysis (n = 20). We also made propensity score for age, sex, and body mass index (PS1) and for all covariables combined (PS2). Statistics were performed using SPSS version 29.0.0.0 (IBM).

## Results

Between April 2019 and May 2024, we included 117 patients referred to our tertiary care center for diagnostic evaluation or initiation of amyloid-specific treatment. The discovery sample comprised 12 patients with ATTR-CM, 5 patients with HF without amyloidosis, and 16 HC subjects. Their demographic characteristics are presented in Supplemental Table 1. The validation sample comprised 86 patients with ATTR-CM, 15 patients with AL affecting the heart, 16 patients with HF, and 23 HC. The demographic characteristics of the validation sample are presented in Table 1.

**Table 1:** Demographic Characteristics of the Validation Cohort

	HC,^a^ (n = 23)	HF,^b^ (n = 16)	ATTR-CM,^c^ (n = 86)	AL,^d^ (n = 15)
Age, y	67 ± 7	69 ± 8^d^	74 ± 9^a^^,^^d^	61 ± 11
Male, n (%)	14 (60.9)	13 (81.3)	76 (88.4)^a^^,^^d^	9 (60)
BMI, kg/m^2^	25.4 ± 4.2	27.4 ± 4.5^a^	25.6 ± 3.3	27.5 ± 3.8^a^
NYHA functional class I/II/III, n		3/6/2	26/47/10	3/5/5^c^
Medical history				
Atrial fibrillation, n (%)	0 (0)	9 (56.3)^d^	51 (59.3)^d^	2 (13.3)
Type 2 diabetes, n (%)	0 (0)	2 (12.5)	9 (10.5)	2 (13.3)
Hypertension, n (%)	0 (0)	5 (31.3)	31 (36)	7 (46.7)
Stroke/TIA, n (%)		1 (6.3)	8 (9.3)	2 (13.3)
Medication				
ACEIs/ARBs, n (%)	0 (0)	7 (43.8)	34 (39.5)	5 (33.3)
Beta-blockers, n (%)	0 (0)	12 (75)	46 (54.1)	7 (46.7)
Loop diuretics, n (%)	0 (0)	2 (12.5)	41 (48.2)	9 (60)^b^^,^^c^
Anticoagulative therapy, n (%)	0 (0)	7 (43.8)	56 (65.1)^d^	4 (26.7)
Antiplatelet therapy, n (%)	0 (0)	8 (50)^c^	17 (19.8)	7 (46.7)^c^
Statins, n (%)	0 (0)	14 (87.5)^c^^,^^d^	45 (52.3)	5 (33.3)
Tafamidis, n (%)	0 (0)	0 (0)	5 (5.8)	0 (0)
Echocardiography				
IVSD, mm		1.3 (1.2-1.8)	1.4 (1.2-1.7)^d^	1.3 (1.0-1.5)
Cardiac output, L/min		4.9 ± 1.1	4.4 ± 1.1	4.6 ± 1.6
LVEF <50, n (%)		2 (14.3)	26 (40.6)	6 (40)
Global longitudinal strain %		−14 ± 2	−11 ± 4	−11 ± 5
LV mass, g		227 (166-265)	250 (208-297)^d^	211 (167-257)
LV mass index, g/m^2^		112 (97-131)	124 (107-150)^d^	99 (83-138)
Hemodynamics				
Pulmonary hypertension, n (%)		7/14 (50.0)	33/50 (66)	11/15 (73.3)
RAP, mm Hg		7 (3-9)	8 (5-11)	9 (7-16)^a^
mPAP, mm Hg		17 (14-30)	24 (19-32)	28 (21-34)^a^
PVR, WU		1.4 (1.2-1.7)	1.7 (1.1-2.2)	1.5 (0.8-2.4)
Biochemistry				
Platelets, ×10^9^/L	269 ± 63^b^^,^^c^	223 ± 54	198 ± 51	239 ± 140^c^
White blood count, ×10^9^/L	5.7 ± 1	6.5 ± 1.9	6.7 ± 1.7^a^	7.4 ± 1.8^a^
Total cholesterol, mmol/L	5.7 (4.8-6.6)^b^^,^^c^^,^^d^	3.8 (3.2-4.4)	4.0 (3.4-4.8)	4.4 (3.8-5.0)
CRP, mg/L	1.3 (0.7-2.1)	1.65 (0.7-4.75)	1.4 (0.7-3.1)	4.25 (2.6-12)^a^^,^^b^^,^^c^
NT-proBNP, ng/L		599 (477-963)	1,562 (739-2,564)	4,841 (2,260-10,985)^b^^,^^c^
cTnt, ng/L		19 (16-35)	43 (33-66)^b^	64 (44-140)^b^^,^^c^
eGFR, mL/min/1.73 m^2^		71 ± 21	68 ± 19	65 ± 29

Values are mean ± SD or median (IQR) unless otherwise indicated.

ACEI = angiotensin-converting-enzyme inhibitor; AL = amyloid light chain; ARB = angiotensin II receptor blocker; ATTR-CM = transthyretin amyloid cardiomyopathy; BMI = body mass index; CRP = C-reactive protein; cTnt = cardiac troponin T; eGFR = estimated glomerular filtration rate; HC = healthy control; HF = heart failure; IVSD = interventricular septum diastolic diameter; LV = left ventricular; LVEF = left ventricular ejection fraction; mPAP = mean pulmonary artery pressure; NT-proBNP = N-terminal pro-brain natriuretic peptide; PVR = pulmonary vascular resistance; RAP = right atrial pressure; TIA = transient ischemic attack.

a, b, c, d *P* < 0.05 vs indicated group.

### Proteomic analysis

**Figure 1:**
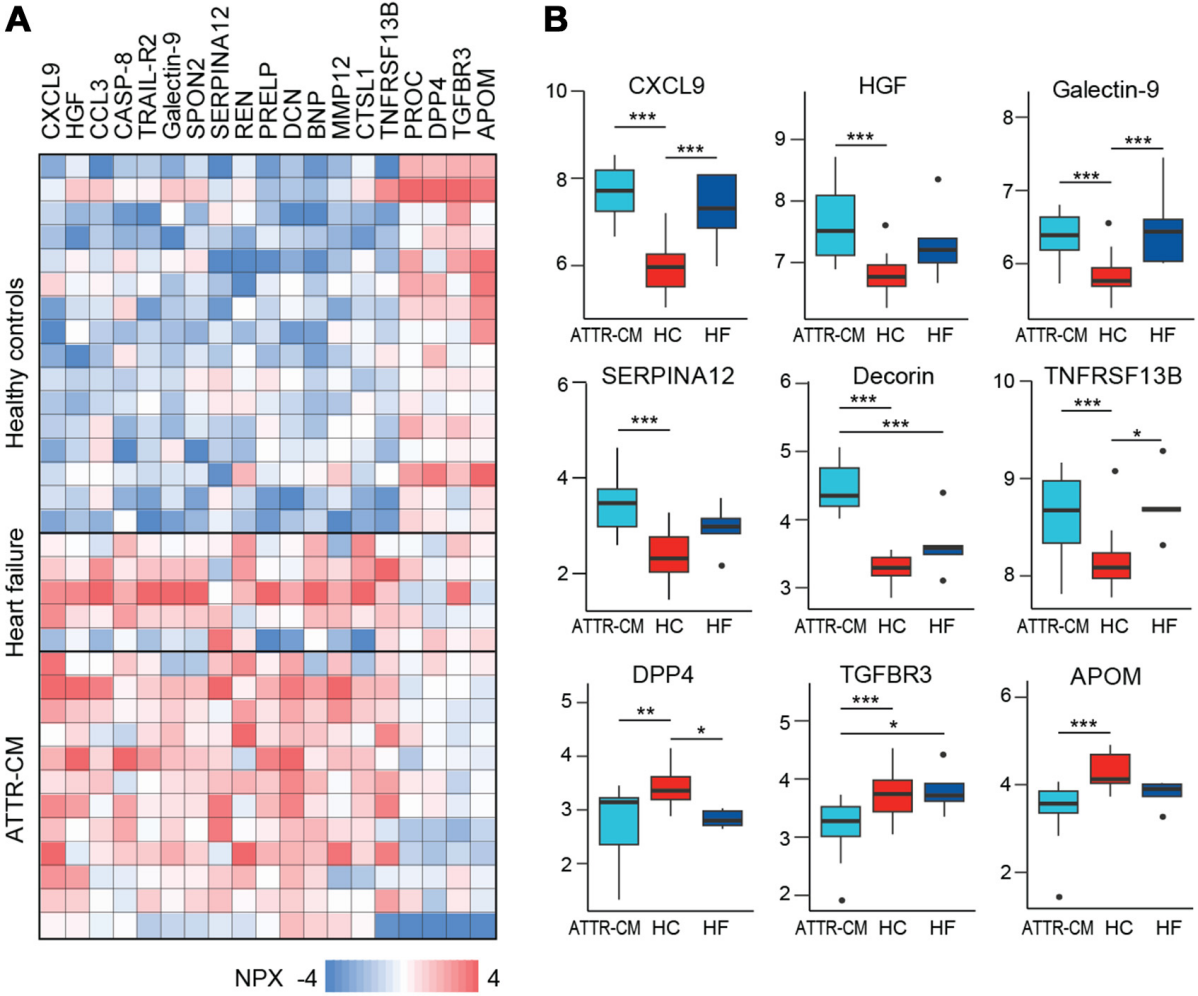
Proteomic Analysis of Plasma in Patients With Transthyretin Amyloid Cardiomyopathy (A) Heatmap showing proteins dysregulated in ATTR-CM compared to patients with HF caused by other medical conditions than ATTR (HF) and HCs. The proteins were quantified using 3 Olink panels; (B) Tukey plots of normalized protein expression levels in ATTR-CM, HF, and HC. NPX, CXCL9, HGF, SERPINA12, DCN, TNFRSF13B, DPP4, TGFβR3, APOM. ∗*P* < 0.01, ∗∗*P* < 0.01, ∗∗∗*P* < 0.001 between indicated groups. Black dots indicate outliers. APOM = apolipoprotein M; ATTR-CM = transthyretin amyloid cardiomyopathy; CXCL9 = C-X-C motif chemokine ligand 9/monokine induced by gamma interferon; DCN = decorin; DPP4 = dipeptidyl peptidase 4; HC = healthy control; HF = heart failure; HGF = hepatocyte growth factor; NPX = normalized protein expression; SERPINA12 = serpin family A member 12; TGFβR3 = transforming growth factor beta receptor 3; TNFRSF13B = tumor necrosis factor receptor superfamily member 13B.

Of 276 proteins assessed across 3 Olink panels, 19 proteins were expressed in different amounts in patients with ATTR-CM compared to patients with HF without amyloidosis and HCs (Figure 1). From these, we selected 9 proteins for further validation analyses based on the measurability and availability of immunoassays. Figure 1B shows normalized protein expression levels from the Olink assays of the 9 selected proteins. Patients with ATTR-CM and patients with HF without amyloidosis had higher levels of CXCL9, galectin-9, and tumor necrosis factor receptor superfamily member 13B (TNFRSF13B) compared with HCs. In contrast, the patients with ATTR-CM or HF had lower levels of DPP4 compared with HCs. Levels of HGF and serpin family A member 12 were higher, and levels of apolipoprotein M were lower, in the patients with ATTR-CM than in HCs. Finally, concentrations of DCN were higher, and concentrations of TGFβR3 were lower, in patients with ATTR-CM compared with HCs and patients with HF without amyloidosis.

### Validation of dysregulated proteins

**Figure 2:**
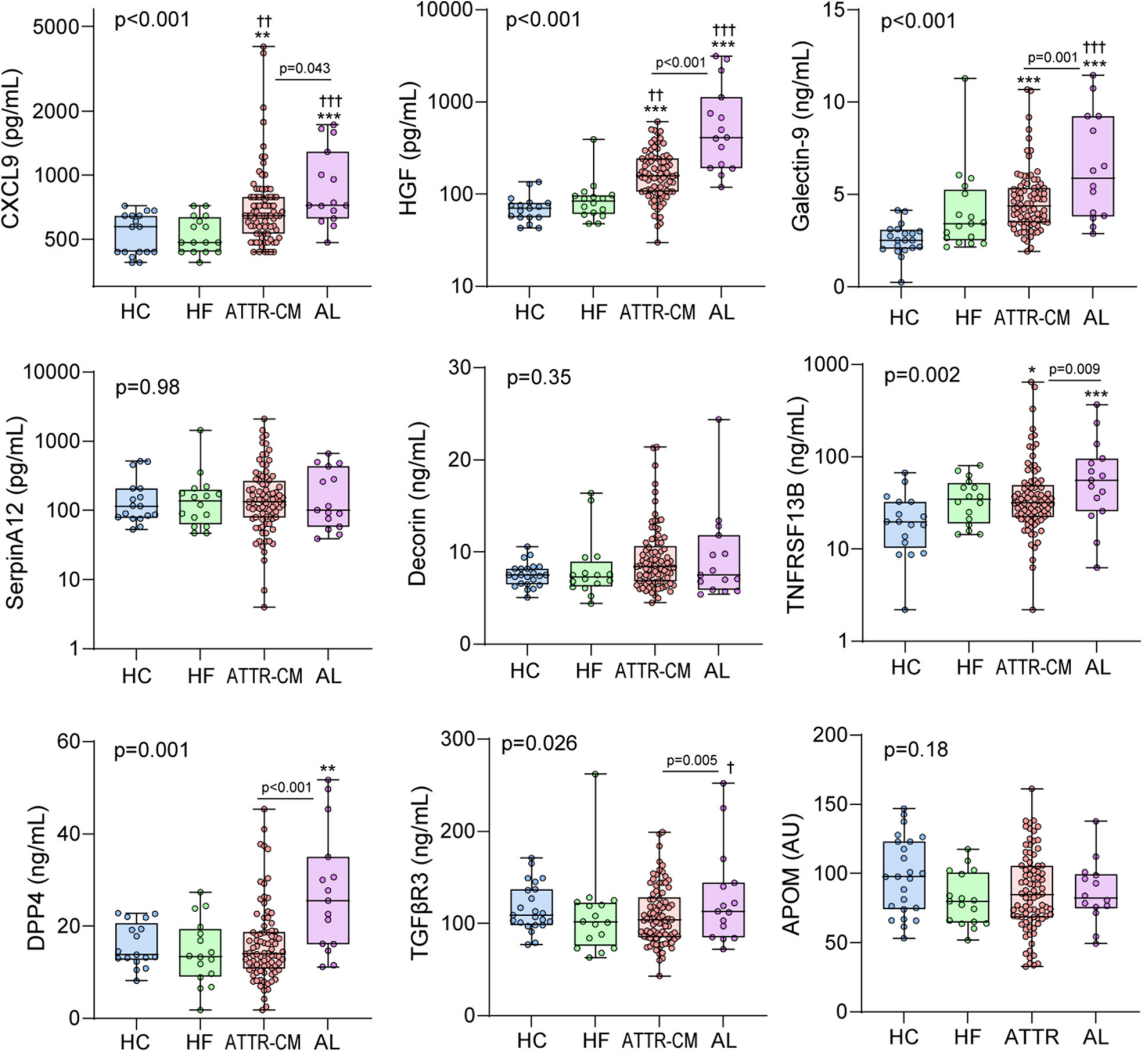
Validation of Dysregulated Proteins in Patients With Transthyretin Amyloid Cardiomyopathy Box-and-whisker plots of proteins assessed in a larger sample of patients with ATTR-CM (n = 86), HCs (n = 23), other HF (n = 16), and AL amyloidosis (n = 15). The proteins were assessed with enzyme immunoassays. CXCL9, HGF, DCN, TNFRSF13B, DPP4, TGFβR3, APOM. The top left *P* value reflects the overall group between-subjects effect from the GLM using age, sex, and BMI as covariates. Post hoc comparisons in the GLM were Sidak-adjusted. ∗*P* < 0.01, ∗∗*P* < 0.01, ∗∗∗*P* < 0.001 vs HC; ^†^*P* < 0.05, ^††^*P* < 0.05, ^†††^*P* < 0.001 vs HF. *P* values comparing ATTR-CM and AL are indicated directly in graphs. AL = amyloid light chain; BMI = body mass index; GLM = general linear model; other abbreviations as in Figure 1.

In the validation sample (Figure 2), circulating levels of CXCL9, HGF, galectin-9, and TNFRSF13B were higher in patients with ATTR-CM than in HCs. Importantly, levels of HGF were also higher in patients with ATTR-CM than in patients with HF without amyloidosis. Levels of CXCL9, HGF, galectin-9, and TNFRSF13B were higher in patients with AL than in HCs. Notably, CXCL9 and HGF were markedly higher in AL than in ATTR-CM. The AL group also had higher levels of DPP4 compared to the other groups. Levels of serpin family A member 12, DCN, and TGFβR3 did not differ significantly across the groups.

### Associations between cardiac function and levels of CXCL9, TNFRSF13B, galectin-9, and HGF

**Figure 3:**
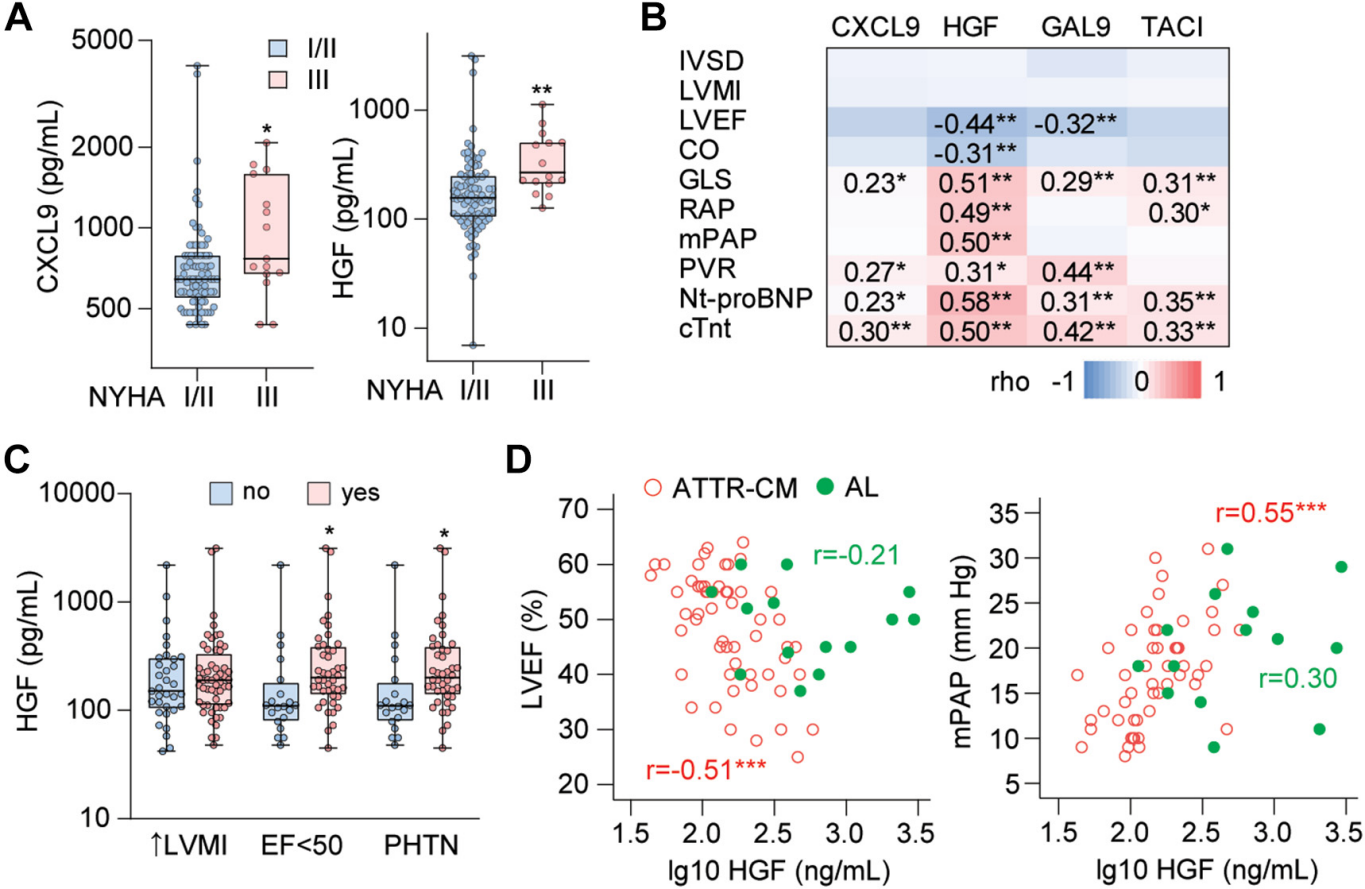
Association Between Dysregulated Proteins and Cardiac Measures in Transthyretin Amyloid Cardiomyopathy and Amyloid Light Chain Amyloidosis (A) Box-and-whisker plots of plasma CXCL9 and HGF in relation to NYHA functional class. ∗*P* < 0.05, ∗∗*P* < 0.01 NYHA functional class III (n = 15) vs NYHA functional class I/II (n = 54) as analyzed by multivariable GLM with age, sex, and BMI as covariables. (B) Heatmap showing correlations (Spearman) between plasma proteins and echocardiographic, hemodynamic, and serologic indices of cardiac function. ∗*P* < 0.05, ∗∗*P* < 0.01. (C) HGF levels according to clinical cutoffs for left ventricular hypertrophy (ie, LVMI above sex-adjusted cutoff^21^); EF below 50%; pulmonary hypertension (PHTN, ie, mPAP >20 mm Hg) ∗*P* < 0.05. (D) Correlation plots showing association between HGF and LVEF (left panel) and mPAP (right panel) in ATTR-CM and AL amyloidosis. ∗∗∗*P* < 0.001. Correlation coefficients (rho) are given in the graphs in ATTR-CM (green) and AL amyloidosis (green). Global longitudinal strain was reported as negative values. Therefore, a positive correlation between the biomarkers and GLS shows that increasing marker levels were associated with poorer left ventricular function. AL = amyloid light chain; ATTR-CM = transthyretin amyloid cardiomyopathy; CO = cardiac output; cTnt = cardiac troponin T; CXCL9 = C-X-C motif chemokine ligand 9/MIG; EF = ejection fraction; GLS = global longitudinal strain; HGF = hepatocyte growth factor; IVSD = interventricular septum diastolic diameter; LVEF = left ventricular ejection fraction; LVMI = left ventricular mass index; mPAP = mean pulmonary artery pressure; NT-proBNP = N-terminal pro-brain natriuretic peptide; PHTN = pulmonary hypertension; PVR = pulmonary vascular resistance; RAP = right atrial pressure.

As shown in Figure 3A, plasma CXCL9 and HGF were higher in patients in NYHA functional class III than in patients in NYHA functional class I or II. A similar trend was observed for TNFRSF13B (*P* = 0.06). HGF and galectin-9 correlated negatively with left ventricular ejection fraction, while CXCL9, HGF, and TNFRSF13B all correlated positively with global longitudinal strain (Figure 3B). Strain was reported as negative values, meaning that high strain values (low absolute values) reflected poor left ventricular function. HGF and TNFRSF13B correlated positively with right atrial pressure and mean pulmonary artery pressure. Finally, all 4 proteins correlated positively with NT-proBNP and cTnT. The associations with measures of cardiac function were stronger for HGF than for the other markers. The association between HGF and clinical cutoffs for left ventricular hypertrophy^21^ and pulmonary hypertension^19^ are shown in Figure 3C, as are levels of HGF stratified by ejection fraction ≥50% vs <50%. Figure 3D shows correlations between HGF and ejection fraction and pulmonary arterial pressure within the ATTR-CM and AL groups. The association between HGF and left ventricular ejection fraction was stronger in ATTR-CM (r = −0.51, *P* < 0.001) than in AL (r = −0.21). Taken together, several of the biomarkers were associated with indices of impaired cardiac function as well as indices of pulmonary hypertension, known characteristics of ATTR-CM. These associations were primarily seen for CXCL9, TNFRSF13B, and HGF.

### Association between CXCL9, HGF, TNFRSF13B, and all-cause mortality

**Figure 4:**
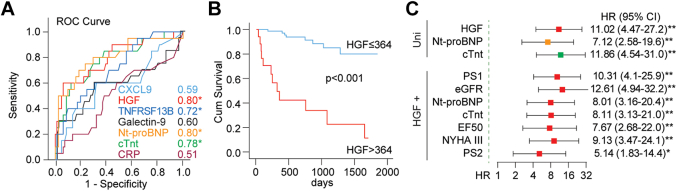
Association Between Dysregulated Proteins and All-Cause Mortality in Transthyretin Amyloid Cardiomyopathy and Amyloid Light Chain Amyloidosis (A) Association between dysregulated proteins (ie, CXCL9, HGF, TNFRSF13B, Galectin-9), cardiac markers (NT-proBNP, cTnt), CRP, and all-cause mortality (n = 20) as assessed by the area under the ROC. Numbers indicate the AUC for different markers. ∗*P* < 0.001. (B) Kaplan-Meier analysis of all-cause mortality according to dichotomized HGF levels (cutoff determined by Youden index). The log-rank *P* value is shown. (C) Cox regression of HGF and all-cause mortality with different levels of adjustment. Uni indicated the unadjusted association between HGF, NT-proBNP, cTnt, and all-cause mortality, while HGF+ indicates models with HGF + one-by-one adjustment with different confounders (PS1 is a propensity score with age, sex, and BMI; PS2 is a propensity score including all confounders: age, sex, BMI, eGFR, NT-proBNP, cTnt, EF50, and NYHA functional class III). ∗*P* < 0.01; ∗∗*P* < 0.001. AUC = area under the curve; BMI = body mass index; CRP = C-reactive protein; cTnt = cardiac troponin T; cTnt = cardiac troponin T; CXCL9 = C-X-C motif chemokine ligand 9/monokine induced by gamma interferon; EF50 = left ventricular ejection fraction <50; eGFR = estimated glomerular filtration rate; HGF = hepatocyte growth factor; NT-proBNP = N-terminal pro-brain natriuretic peptide; ROC = receiver-operating characteristics curve; TNFRSF13B = tumor necrosis factor receptor superfamily member 13B.

During a median follow-up of 637 days (range 30-1,872 days), 13 patients with ATTR-CM and 7 patients with AL died. Due to the limited number of events, associations between the biomarkers and all-cause mortality are reported for patients with ATTR-CM and AL combined. As shown in Figure 4A, TNFRSF13B had a modest discriminatory ability to predict all-cause mortality. On the other hand, the discriminatory ability for HGF was good at an area under the curve of 0.80 (*P* < 0.001). The ability of the latter to distinguish between patients who died and patients who did not was similar to those of NT-proBNP and cTnT. Figure 4B shows survival stratified by HGF levels above or below 364 pg/L. High levels of TNFRSF13B were also associated with poor outcomes (Supplemental Figure 1A). In contrast, CXCL9 was not associated with mortality in CA.

The hazard rate for all-cause mortality for HGF above the optimal cut-point of 364 pg/L was 11.0 (Figure 4C). For TNFRSF13B, the corresponding HR was 12.6 (Supplemental Figure 1B), but the CIs were wide. For comparison, the HRs for NT-proBNP and cTnT were 7.1 and 11.9, respectively. The association between high levels of HGF and all-cause mortality was attenuated by adjustment for other predictors of cardiac death, but remained highly significant (bottom part of Figure 4C). Similarly, the association between TNFRSF13B and all-cause mortality remained significant on adjusted analysis.

We also assessed whether combinations of markers would increase discrimination of all-cause mortality by comparing precision-recall curves. As shown in Supplemental Figure 2, recall was approximately 40% to 60% for all markers and combinations. However, the levels of precision differed. For both cTnT and NT-proBNP, discrimination increased significantly when combined with HGF compared with that of either marker alone. Levels above the optimal cut-point of both NT-proBNP and HGF identified 50% of patients who died, and approximately 85% were true positives.

## Discussion

In this single-center study, proteomic screening identified 9 proteins that are dysregulated in ATTR-CM. Validation in a larger patient sample revealed that CXCL9, TNFRSF13B, galectin-9, and HGF were higher in patients with ATTR-CM than in HCs. In addition, levels of CXCL9 and HGF were higher in patients with ATTR-CM than in patients with HF without amyloidosis. Levels of these markers were higher in AL than in ATTR-CM. In patients with ATTR-CM or AL, high levels of these markers, and particularly HGF, were associated with echocardiographic and hemodynamic measures of poor cardiac function. Finally, high levels of HGF or TNFRSF13B were independently associated with all-cause mortality.

Degenerated monomers of transthyretin and light-chain proteins display amylogenic motives in the form of beta-pleated sheets that, under certain conditions, combine to form oligomers. These oligomers can polymerize in an energetically favorable process that produces insoluble amyloid fibrils. However, what precipitates this process and the mechanism behind the affinity for cardiac tissue is not known. AL and ATTR-CM are both associated with interstitial fibrosis.^22^ HGF is a multifunctional cytokine secreted by mesenchymal cells. It is expressed in HF and postmyocardial infarction, promoting scarless wound healing.^23^ The extracellular pathology in CA may induce HGF as a counter-responsive measure.

Other groups have also found that levels of HGF are elevated in patients with CA. Abraham et al^24^ showed that circulating concentrations of HGF were substantially higher in patients with AL than in patients with monoclonal gammopathy of undetermined significance. They also found a positive correlation between HGF and the degree of cardiac involvement. Zhang et al^25^ reported that HGF levels were higher in patients with AL (n = 43) or ATTR-CM (n = 29) compared to patients with HF with reduced ejection fraction or with left ventricular hypertrophy but without CA. High levels of HGF discriminated between patients with CA and patients with HF with reduced ejection fraction or hypertrophy, and were associated with worse outcomes.^25^

In our study, we extend and confirm these findings in several ways. First, we confirm the link between HGF and ATTR-CM in a larger cohort of patients. Second, we show that levels of HGF are higher in CA than in HF caused by other etiologies (Central Illustration). Third, HGF correlates with multiple indices of poor cardiac function, suggesting that HGF might be involved in the progression of the disease. Left ventricular hypertrophy occurs early in CA, whereas left and right ventricular systolic dysfunction develop later.^26^ We found that HGF correlated poorly with left ventricular mass, but strongly with systolic and diastolic dysfunction.

**Central illustration:**
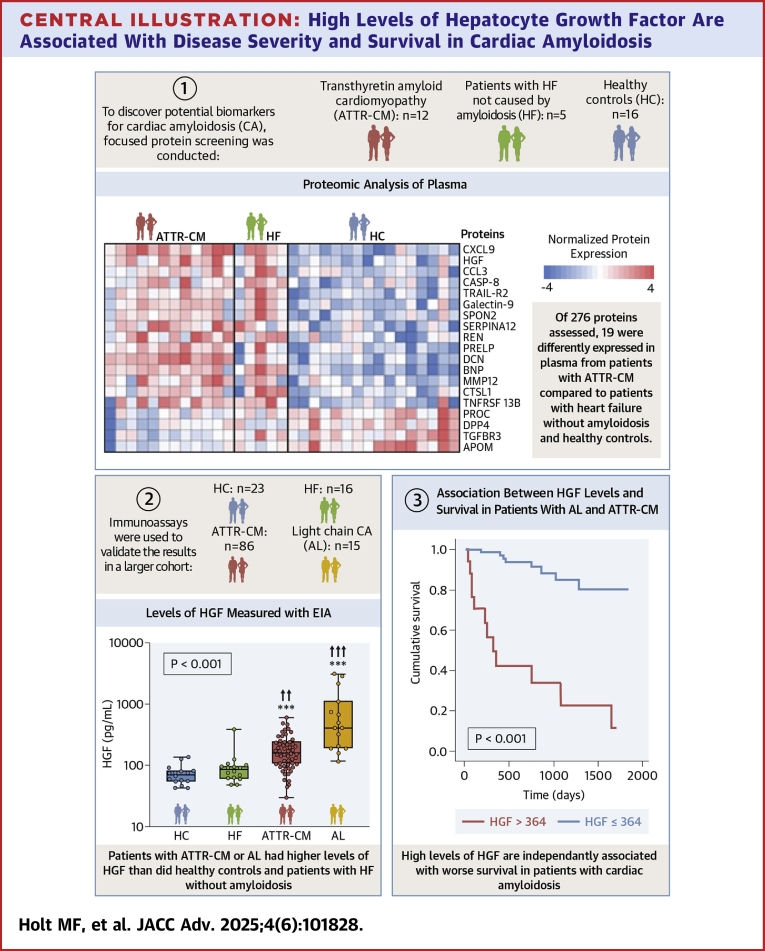
High Levels of Hepatocyte Growth Factor Are Associated With Disease Severity and Survival in Cardiac Amyloidosis We used targeted proteomics to discover potential biomarkers in patients with cardiac amyloidosis. To validate the result, we evaluated the regulated proteins in a larger cohort, using immunoassays. Levels of hepatocyte growth factor were significantly higher in patients with cardiac amyloidosis than in patients with HF without amyloidosis and HCs. High levels of HGF were associated with echocardiographic and hemodynamic measures of poor cardiac function. High levels of HGF were independently associated with all-cause mortality. AL = amyloid light chain; HC = healthy control; HF = heart failure; other abbreviations as in Figure 1.

Finally, we found that high HGF levels were independently associated with poorer survival in patients with CA (Central Illustration). These results are in line with those of Zhang et al,^24^ who found that HGF was associated with cardiac outcomes and improved predictive models that included cTnT and NT-proBNP. The available data on HGF in patients with CA suggest that HGF plays a particularly salient role in amyloid heart disease. However, high HGF levels are associated with increased risk of HF in the general population.^4^ Furthermore, HGF is a strong and independent predictor of all-cause mortality in patients with acute^27^ and advanced^28^ systolic HF. The proportion of patients with CA in these studies is unknown, but the studies suggest that HGF is a marker for HF in general rather than for CA specifically. HGF is proposed to have cardioprotective effects through the activation of pathways that enhance angiogenesis and limit apoptosis, inflammation, and fibrosis.^29^ High levels of HGF are associated with concentric left ventricular remodeling.^30^ Activation of the receptor of HGF, Met, induces concentric cardiac hypertrophy in experimental models.^31^

Except for HGF, CXCL9 was the only biomarker that was significantly higher in patients with ATTR-CM than in patients with HF of other etiologies. CXCL9 induces chemotaxis and is associated with the development of HF through largely unknown mechanisms.^32^ We found no previous studies linking CXCL9 to CA. However, CXCL9 promotes cardiac fibrosis through effects on proliferation and migration of fibroblast activity.^33^ CXCL9 could play a role in the inflammatory response to amyloid accumulation through chemotaxis of activated T cells in cardiac tissue.^34^ T-lymphocytes and macrophages are present in amyloid deposits and may contribute to tissue damage.^35^

Proinflammatory cytokines, including tumor necrosis factor, are consistently elevated in HF.^36^ Tumor necrosis factor receptor superfamily member 13 partakes in the tumor necrosis factor signaling cascade. It has been implicated in the development of pulmonary hypertension,^37^ HF,^38^ and AL.^39^ However, the mechanisms by which TNFRSF13B is associated with outcomes in CA remain to be determined. To the best of our knowledge, we are the first to explore levels of TNFRSF13B in CA. Based on its strong association with mortality, TNFRSF13B should be further investigated in CA.

Very recently, Tubben et al^40^ presented data on ATTR-CM in a discovery sample and a larger validation cohort. In contrast to our results, they found that the proteoglycan DCN, lysosomal hydrolase alpha-L-iduronidase, and glycosyl hydrolase galactosidase β-1 most effectively characterized patients with ATTR-CM. In our study, plasma levels of DCN were higher in ATTR-CM than in patients with HF from other causes, but this was not verified in the larger validation sample. Importantly, the study by Tubben et al comprised patients with left ventricular ejection fraction >40% only, most of whom had preserved ejection fraction.

### Study Limitations

Our study has several limitations. First, all patients were included at a tertiary care referral center, and referral biases could limit the applicability of our results to a broader setting. NYHA functional class and echocardiography are subject to observer bias. Furthermore, patients were included at different disease stages, which may influence the results. The study does not provide outcome data besides all-cause mortality. Deaths may not be related to the cardiac disease per se. The limited number of patients with HF without amyloid etiology prevented comparison of the discriminatory capacity of HGF between patients with CA and patients with HF without CA. Several markers differed markedly between patients with ATTR-CM and the other subjects in the screening cohort, but not in the larger validation cohort. The discrepancy between the 2 cohorts emphasizes the importance of revisiting results from small studies in larger samples. Although this is one of the larger studies to compare inflammatory biomarkers between patients with AL or ATTR-CM, patients with HF, and HCs, the number of subjects was limited, and some of the findings may be incidental. Lastly, as with any blood biomarker, circulating levels may not be reflective of what is happening in the myocardium. Studies on myocardial tissue are needed to further understand the pathology of CA.

## Conclusions

Circulating levels of HGF are associated with CA and with the degree of disease severity and mortality in CA. HGF is a potential screening tool to reduce diagnostic delay and select patients for more aggressive treatment. Furthermore, the plasma concentration of HGF offers independent information about survival in patients with CA and could be a supplement to the conventional prognostic biomarkers in this disease.

Perspectives **COMPETENCY IN MEDICAL KNOWLEDGE:** Patients with CA have higher levels of HGF than patients with HF without amyloidosis and HCs. Levels of HGF are associated with disease severity in patients with CA. Plasma levels of HGF provide additional information about survival in patients with CA and could be a supplement to conventional cardiac biomarkers.

**TRANSLATIONAL OUTLOOK:** Circulating levels of HGF may not be reflective of what is happening in the myocardium of patients with CA. Further studies are needed to clarify the pathophysiological explanation of HGF elevation in patients with CA.

## Funding support and author disclosures

The study was in part funded by a grant from The Bergesen Foundation and by a grant from The Blix Family Foundation to Dr Flesvig Holt. The study was in part funded by an unrestricted grant from Pfizer to Dr Gude. The study was in part funded by the K.G. Jebsen Centre for Cardiac Research. The funders were not involved in the collection, analysis, or interpretation of data; the writing of this article; or the decision to submit it for publication. Dr Gullestad has received lecture fees from AstraZeneca, Boehringer Ingelheim, and Novartis and has sat on advisory boards for AstraZeneca and Boehringer Ingelheim. Dr Broch has received lecture fees and consulting fees from Pfizer and has sat on advisory boards for AstraZeneca, Pharmacosmos, Boehringer Ingelheim, and Pfizer. Dr Gude has received grants and honoraria for lectures from Pfizer, Boehringer Ingelheim, and Novartis and has sat on advisory boards for Pfizer. Dr Wien has received lecture fees from Pfizer and Janssen-Cilag and has sat on advisory boards for Alnylam. All other authors have reported that they have no relationships relevant to the contents of this paper to disclose.
