# Supplementary material for: Hepatocyte Growth Factor: A Marker of Cardiac Function, Mortality, and Disease Subtype in Cardiac Amyloidosis
Source: JACC Adv. 2025 Jun 25;4(6):101828. doi: 10.1016/j.jacadv.2025.101828 (PMC12287948; doi:10.1016/j.jacadv.2025.101828)
Supplement: Supplementary data [file mmc1.pdf]

Supplemental material:

Supplemental table 1. Demographic characteristics of the discovery cohort

	HC<br>n = 16	HF<br>n = 5	ATTR-CM<br>n = 12	p-values
Age, years	60.4 ± 7.7	73.8 ± 1.6	73.3 ± 7.2	<0.001
Male, n (%)	8 (50)	3 (60)	9 (75)	0.407
BMI, kg/m^2	25.8 ± 3.6	27.1 ± 6.8	26.0 ± 3.7	0.838
Medical history				
Atrial fibrillation, n (%)	0 (0)	2 (40)	7 (58.3)	0.002
Type 2 diabetes, n (%)	0 (0)	1 (20)	4 (33.3)	0.241
Hypertension, n (%)	0 (0)	0 (0)	5 (41.7)	0.006
Biochemistry				
Hemoglobin, g/dL	14.3 ± 1.1	12.1± 2.6	14.0 ± 1.4	0.068
Creatinine, mg/dL	71.7 ± 12.8	125.2 ± 40.6	101.8 ± 29.8	<0.001
Total cholesterol, mmol/L	5.7 ± 0.8	4.0 ± 0.8	4.1 ± 0.9	<0.001
LDL cholesterol, mmol/L	3.6 ± 0.8	2.1 ± 0.7	2.4 ± 0.8	<0.001
HDL cholesterol, mmol/L	1.8 ± 0.5	1.4 ± 0.4	1.4 ± 0.3	0.157
CRP, mg/L	1.5 ± 1.4	6.1 ± 6.8	2.5 ± 2.7	0.026

HC, healthy controls; HF, heart failure; ATTR-CM, transthyretin amyloid cardiomyopathy; BMI, Body mass index; LDL, low-density lipoprotein; HDL, high-density lipoprotein; CRP, C-reactive protein.

Supplemental Figure 1 - Association between TNFRSF13B and All-cause mortality in patients with cardiac amyloidosis

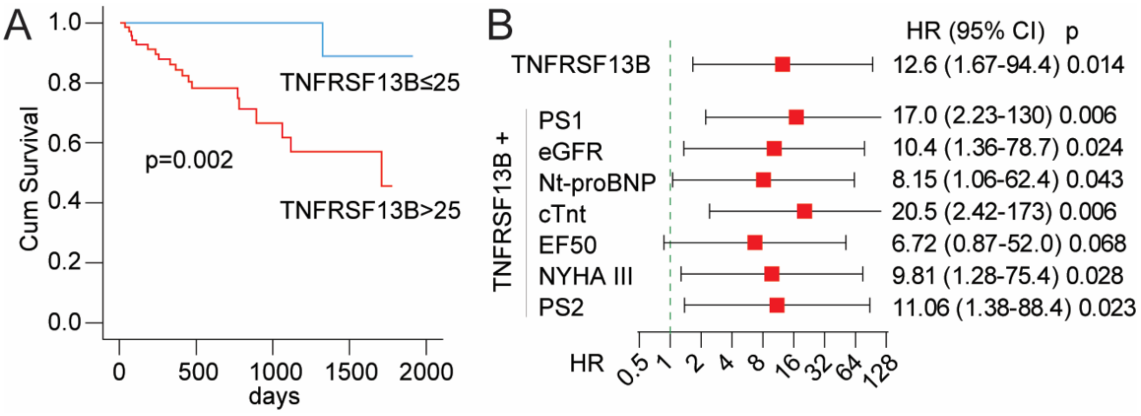

Supplemental Figure 1. Association between TNFRSF13B and All-cause mortality in transthyretin amyloid cardiomyopathy (ATTR-CM) and AL amyloidosis. A) Kaplan–Meier analysis of all-cause mortality according to dichotomized TNF Receptor Superfamily Member 13B (TNFRSF13B/TACI) levels (cut-off determined by Youden's index). Log-rank p-value is shown. **B)** Cox regression of TNFRSF13B and all-cause mortality with different levels of adjustment. TNFRSF13B indicated univariable analysis while TNFRSF13B + indicates models with TNFRSF13B + one-by-one adjustment with different confounders (PS1 is a propensity score with age, sex and BMI; PS2 is a propensity score including all confounders: age, sex, BMI eGFR, NT-proBNP, cTnt, EF50, NYHA III). *p<0.01; **p<0.001. eGFR, estimated glomerular filtration rate; NT-proBNP, N-terminal pro-brain natriuretic peptide; cTnt, Cardiac troponin T; EF50, Left ventricular ejection fraction <50; NYHA, New York Heart Association classification.

Supplemental Figure 2 - Precision-Recall curves for discriminating all-cause mortality in patients with cardiac amyloidosis combining different biomarkers

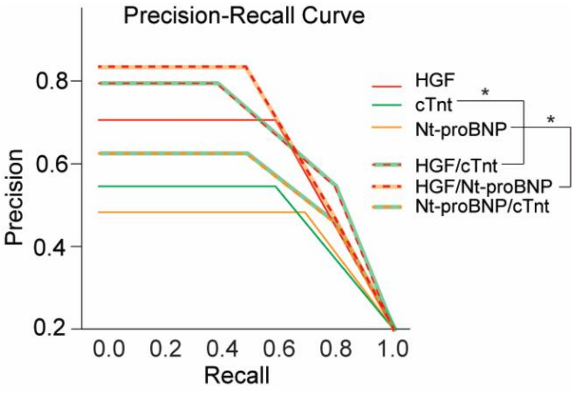

Supplemental Figure 2. Precision-Recall curves for discriminating all-cause mortality in transthyretin amyloid cardiomyopathy (ATTR-CM) and AL amyloidosis using combination of different biomarkers. Cut-offs for all-cause mortality were determined by ROC analysis: HGF: 364 pg/mL, cTnt: 78 ng/L, NT-proBNP: 2750 ng/mL. NT-proBNP, N-terminal pro-brain natriuretic peptide; cTnt, Cardiac troponin T. *p<0.05.
